# European elms as potential hosts of *Saperda tridentata* (Coleoptera: Cerambycidae)

**DOI:** 10.1093/ee/nvag094

**Published:** 2026-09-08

**Authors:** Giacomo Santoiemma, Jon Sweeney, Kate Van Rooyen, Deepa S Pureswaran, Andrea Battisti, Alberto Santini, Francesco Pecori, Davide Rassati

**Affiliations:** Department of Agronomy, Food, Natural Resources, Animals and Environment (DAFNAE), University of Padova, Legnaro, Padova, Italy; Natural Resources Canada, Canadian Forest Service, Atlantic Forestry Centre, Fredericton, New Brunswick, Canada; Natural Resources Canada, Canadian Forest Service, Atlantic Forestry Centre, Fredericton, New Brunswick, Canada; Natural Resources Canada, Canadian Forest Service, Atlantic Forestry Centre, Fredericton, New Brunswick, Canada; Department of Agronomy, Food, Natural Resources, Animals and Environment (DAFNAE), University of Padova, Legnaro, Padova, Italy; Institute for Sustainable Plant Protection (CNR-IPSP), National Research Council, Sesto Fiorentino, Firenze, Italy; Institute for Sustainable Plant Protection (CNR-IPSP), National Research Council, Sesto Fiorentino, Firenze, Italy; Department of Agronomy, Food, Natural Resources, Animals and Environment (DAFNAE), University of Padova, Legnaro, Padova, Italy

**Keywords:** Cerambycidae, feeding, development, wood-borers, invasions

## Abstract

The elm borer, *Saperda tridentata* (Olivier, 1795) (Coleoptera: Cerambycidae), is a North American longhorn beetle that has been repeatedly intercepted in Europe on imported elm logs. Although climatic conditions are considered suitable for its establishment, uncertainty remains regarding the suitability of European tree species as hosts. We evaluated the potential host range of *S. tridentata* by testing 4 elm species (*Ulmus glabra*, *Ulmus laevis*, *Ulmus minor*, and *Ulmus pumila*), 3 maple species (*Acer campestre*, *Acer platanoides*, and *Acer pseudoplatanus*), and 3 poplar species (*Populus alba*, *Populus nigra*, and *Populus tremula*) that are widespread in Europe. No-choice and choice feeding bioassays were conducted to assess adult feeding preference, while no-choice development bioassays were performed to determine whether larvae could establish and develop within logs of the tested species. Adults fed on all elm species but showed no feeding activity on maples or poplars. Feeding levels on European elms did not differ significantly from those recorded on the primary North American host, *U. americana*. Larvae were recovered from logs of all 4 European elm species, confirming their suitability for larval establishment, whereas no larvae were found in maple or poplar logs. Adult emergence was limited, with only 3 individuals emerging from *U. minor* logs. Our results indicate that European elm species can support both adult feeding and larval development of *S. tridentata*, whereas the tested maple and poplar species appear to be unsuitable hosts. These findings suggest that host availability is unlikely to limit the establishment of *S. tridentata* in Europe if introductions occur.

## Introduction

Longhorn beetles (Coleoptera: Cerambycidae) represent one of the most species-rich beetle families worldwide, comprising more than 35,000 described species (Švácha and Lawrence 2014). Beyond their crucial ecological role in forest ecosystems (Haack 2017), longhorn beetles have recently attracted considerable attention in invasion science due to their frequent interception in untreated wood packaging materials, roundwood, and live plants (Eyre and Haack 2017, Meurisse et al. 2019). Once introduced into new environments, some longhorn beetles became invasive, causing substantial economic and ecological damage to trees in both urban and forest habitats (Haack 2017). For instance, *Anoplophora glabripennis* (Motschulsky, 1854) is ranked among the top 10 invasive species (including weeds and vertebrates) in terms of post-invasion management costs (Cuthbert et al. 2022). Despite ongoing phytosanitary efforts aimed at reducing international insect incursions (Allen et al. 2017, Nahrung et al. 2023), the risk of non-native longhorn beetle introductions persists (Ruzzier et al. 2020, Coyle et al. 2021, Mondaca et al. 2023, Lee et al. 2024).

The elm borer *Saperda tridentata* (Olivier, 1795) is a longhorn beetle native to North America. It is widespread in deciduous forests of the eastern United States and southern Canada (Bousquet et al. 2013, Monné and Nearns 2023), and its distribution largely overlaps with that of American elm species (*Ulmus* spp.). *S. tridentata* is a univoltine species. Larvae overwinter beneath the bark and in the sapwood, whereas newly emerged adults require a short maturation feeding period and can mate after approximately 3 to 4 days of feeding (Pechuman 1940, EFSA et al. 2020). Females preferentially oviposit on stressed, declining, or recently weakened trees (Campbell et al. 1989).

In the last decade, larvae of *S. tridentata* have been intercepted multiple times in Italy on red elm (*Ulmus rubra* Muhl.) logs with bark originating from various states in eastern North America (Binazzi et al. 2019, EFSA et al. 2020). These recurrent interceptions have led European plant health authorities to evaluate its establishment potential in the EU and the associated risks to European tree species (EFSA et al. 2020, EPPO 2021). Current analyses suggest that establishment would not be limited by climatic conditions, as climates in the North American range of *S. tridentata* are similar in Europe (EFSA et al. 2020, Ruzzier et al. 2026). In contrast, the role of host availability in the EU and the potential impacts on European tree species remain uncertain, largely due to the limited information on host species suitability.

*S. tridentata* has been reported to develop primarily on *Ulmus americana* L., although *U. rubra* has also been recognized as a suitable host (Felt and Joutel 1904, Haack 2021). *Ulmus crassifolia* Nutt. has likewise been proposed as a host (Solomon 1995). Additionally, adults have been recorded on *Ulmus alata* Michx. (winged elm), a species native to the south-central and southeastern United States (MacRae 1993); however, its suitability as a reproductive host remains uncertain. All of these *Ulmus* species are present in Europe, but their distribution is generally limited, with occurrences largely confined to arboreta and likely some private gardens (EFSA et al. 2020). Thus, *S. tridentata* is unlikely to establish in Europe if these North American elms are the only suitable hosts. The likelihood of establishment would increase substantially if *S. tridentata* were able to reproduce on elm species that are widespread in Europe. *Ulmus glabra* Huds., *Ulmus minor* Mill., *Ulmus laevis* Pall., and *Ulmus pumila* L. are common across much of Europe (Caudullo and de Rigo 2016), even though their populations were strongly affected by the Dutch elm disease (Santini and Faccoli 2015). Nonetheless, there is no observational evidence that *S. tridentata* can attack and develop on these elm species. A few old and scattered reports also mentioned larvae in non-elm genera, such as maples (*Acer* spp.) and poplars (*Populus* spp.) (Ehrmann 1897, Washburn 1910), although these observations remain anecdotal (EFSA et al. 2020). Clarifying whether European elm, maple, and poplar species may be hosts for *S. tridentata* would aid in assessing its potential establishment and impact in Europe.

In this study, we investigated the potential suitability of European tree species belonging to the genera *Ulmus* (4 species), *Acer* (3 species), and *Populus* (3 species) as hosts for *S. tridentata*. Specifically, we conducted: (i) no-choice feeding bioassays to assess the ability of *S. tridentata* adults to feed on leaves of the tested European species; (ii) choice feeding bioassays to evaluate preferences between its primary host in the native range, *U. americana*, and the tested European species; and (iii) development bioassays to determine whether the tested species can support larval development and completion of the life cycle. In addition to expanding existing ecological knowledge of this longhorn beetle species, the results of this study provide relevant information to assess the invasion potential of *S. tridentata* in the European Union.

## Materials and Methods

### Collection and Shipment of Logs and Saplings of European Tree Species

Saplings for feeding bioassays and logs for development bioassays of European elm, maple, and poplar species were sourced in Italy and shipped to the Atlantic Forest Centre (AFC) in Fredericton, New Brunswick, Canada, following the receipt of specific phytosanitary permits. The process to obtain these permits from the Canadian Food Inspection Agency (CFIA) began in December 2022; permits were granted in March 2023 and subsequently renewed in March 2024.

Logs (approximately 8 to 15 cm in diameter and 40 cm in length) were obtained in January and February 2023 by cutting trees and branches from standing trees located at various sites in the Tuscany region, Italy. Ten logs were collected for each of the following tree species: *Acer campestre* L., *Acer platanoides* L., *Acer pseudoplatanus* L., *Populus alba* L., *Populus nigra* L., *Populus tremula* L., *U. glabra*, *U. laevis*, *U. minor*, and *U. pumila*. Immediately after cutting and prior to shipment, both ends of each log were sealed with wax to prevent desiccation. Most logs were shipped in March 2023; however, logs of *U. laevis* and *U. pumila* were obtained and shipped in January 2024 due to difficulties in locating suitable source trees in 2023.

Twenty saplings (approximately 1 cm in stem diameter and 1.5 m in height) of each of the 10 species were purchased from a nursery in Tuscany (Vannucci Piante, Pistoia, Italy) and shipped bare-root together with the logs in March 2023. Bare-root shipment was preferred to reduce the phytosanitary risk associated with potted plants, even when asymptomatic (Migliorini et al. 2015). Upon arrival in Canada, saplings were immediately transplanted into pots and maintained in containment facilities at the AFC. Logs were stored in sealed plastic Rubbermaid tubs and maintained in a containment freezer cell at −12 °C until use.

### Collection of *S. tridentata* Adult Beetles

**Fig. 1.**
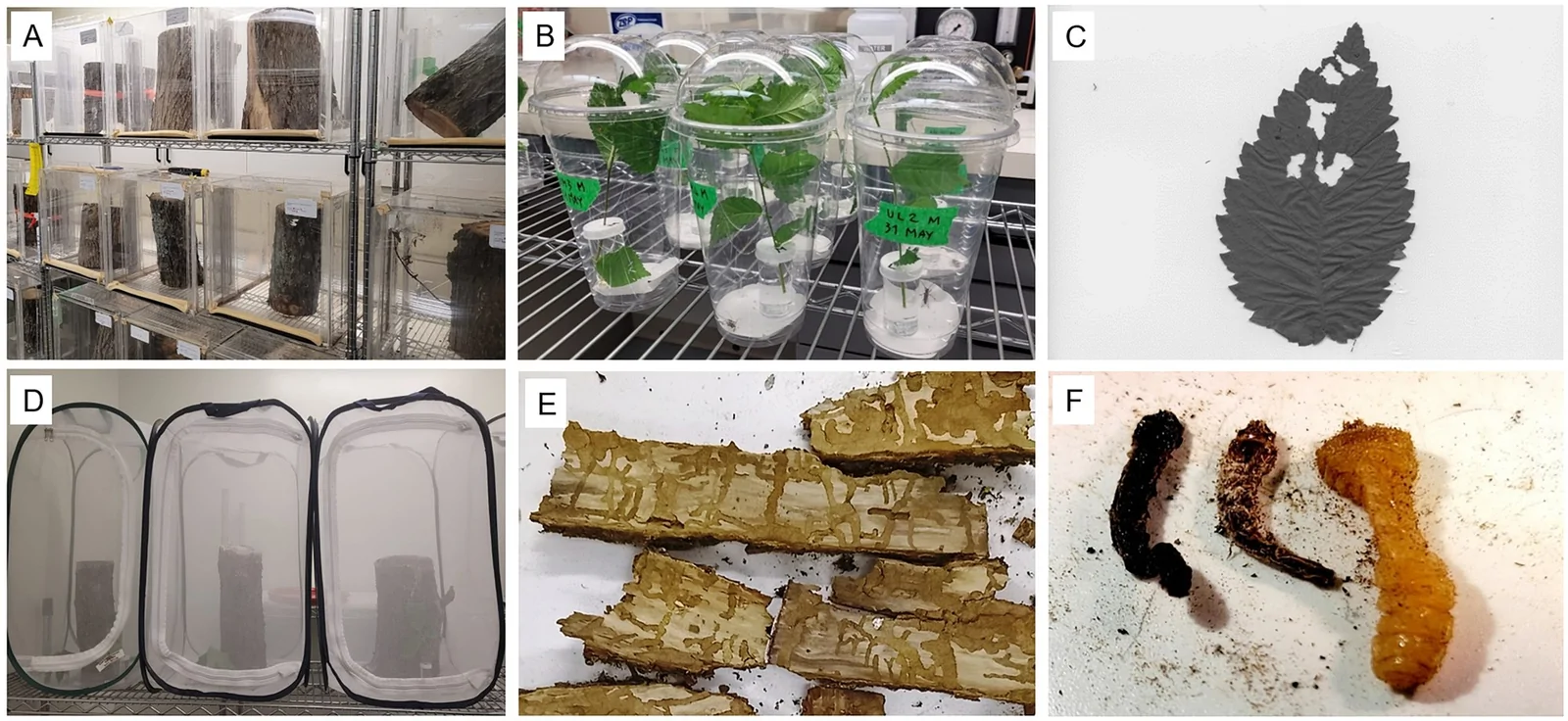
(A) *U. americana* logs arranged within hermetic cages to monitor the emergence of adult *S. tridentata*; (B) plastic cups in which *S. tridentata* adults were confined together with a leaf branch of one of the target tree species; (C) leaf scanned image analyzed using the software WinFOLIA to estimate the leaf area consumed; (D) logs enclosed within tulle cages in which a pair of adult beetles (1 male and 1 female) was released; (E) *U. minor* bark pieces showing larval galleries; (F) dead larvae found within the bark.

Adult *S. tridentata* beetles were obtained from field-collected logs. Specifically, potentially infested logs (∼30 to 40 cm in diameter and 50 cm in length) were collected biweekly in Fredericton during spring–summer 2023 (April to August), and winter–spring 2024 and 2025 (January to April) from dying *U. americana* trees that the City of Fredericton had felled to reduce the spread of Dutch elm disease. *U. americana* logs were transferred to the AFC rearing facility and maintained at about 20 to 24 °C and 30% to 60% RH. Each log was placed individually in a sealed emergence cage (Fig. 1A) and inspected 5 to 7 days per week for adult emergence. Newly emerged beetles were collected for subsequent feeding and development bioassays. Following the same protocols, preliminary collection and incubation of 25 bolts from these trees in 2022 produced a total of 281 adult *S. tridentata* (139 males, 142 females) with an average of 11.2 ± 3.4 adults emerging per bolt.

### Feeding Bioassays

Two types of feeding bioassays, no-choice and choice, were conducted. For both experiments, branches were collected from the European saplings representing the 10 tested species, as well as from *U. americana* (control), either sampled from plants grown near the AFC in June 2023 or obtained from greenhouse-grown plants in March 2024. In both cases, 1 branch bearing 1 or more leaves was collected from each species so that all samples had a similar total leaf area, obtained either from the combined area of multiple leaves or from a single larger leaf. No-choice bioassays were performed from 16 May to 20 June 2023 by confining a single adult *S. tridentata* in a plastic cup (710 ml) with a branch of a target tree species (Fig. 1B). Cups were ventilated through perforated lids and covered with fine metal mesh to prevent beetle escape. Branches were placed in small water-filled containers to maintain leaf turgor, and cups were lightly sprayed with water daily to prevent desiccation. For each of the 10 European tree species, 10 replicates were performed. Only for *U. americana*, used as a control, 12 replicates were conducted. Branches were roughly evenly split between females and males (generally 5 of each).

Choice feeding bioassays were carried out from 13 June to 5 July 2023 and from 14 to 21 February 2024. The trials followed the same protocol described for the no-choice tests. The only difference was that 2 branches were placed in each plastic cup: 1 from *U. americana* (control) and 1 from 1 of the 10 European tree species. Thus, each replicate consisted of a paired comparison between *U. americana* and a single European species. Twelve replicates were performed for each European species, with branches mostly evenly split between females and males (generally 6 of each).

Feeding activity was estimated by measuring the total leaf area of each branch (mm²) before beetle release and after 24 and 48 hours of exposure (Fig. 1C). Leaves were scanned and analyzed using WinFOLIA software (Regent Instruments Inc., Quebec, Canada; https://regent.qc.ca/assets/winfolia_software.html) to quantify the area consumed by beetles at each time point. This method has been previously used to assess beetle feeding behavior on different host species (Pureswaran and Poland 2009).

Body size was assessed on retained specimens from the feeding bioassays by measuring elytra length and maximum thoracic width in 10 males and 10 females.

### Development Bioassays

A no-choice experiment was conducted to assess the ability of *S. tridentata* to develop on logs of the 10 selected European tree species. Development was evaluated based on the presence of larval galleries, the number of larvae, and the emergence of adult beetles. Prior to the main experimental trials, preliminary trials in 2022 using *U. americana* logs were carried out to test the experimental method, adapting a protocol used by Flaherty et al. (2011) for rearing brown spruce longhorn beetle, *Tetropium fuscum* (Fabricius, 1787), on spruce bolts. Eleven bolts were cut from branches of live *U. americana* trees in Fredericton, New Brunswick. The bolts averaged 45 (40 to 49) cm in length and fell into 4 diameter size classes: 5 to 8.9 cm (3 bolts), 9 to 10.9 cm (2 bolts), 11 to 15.5 cm (2 bolts), and >15.5 cm (4 bolts). All bolts in each of the 2 smaller size classes were housed together in 2 separate Lexan cages (46 × 46 × 46 cm) grouped by size class whereas each of the 6 bolts in the 2 larger diameter classes were housed individually in separate Lexan cages. The bolts were incubated at 20 to 24 °C, 30% to 60% RH, and a 16:8 L: D cycle. We placed 3 *S. tridentata* mating pairs in each cage and provided them with fresh American elm foliage that was replaced as needed. The beetles were allowed to mate and lay eggs for their entire adult life. After all beetles had died, we waxed the ends of the bolts with paraffin to reduce desiccation and kept them in the same conditions for 3.5 months to allow for larval development. We then incubated them at 5 °C for 2 weeks followed by 3 months at −9 °C, after which we set the bolts up at 20 °C and checked for adult emergence 5 days per week for 12 weeks. A total of 65 adult *S. tridentata* (43 males, 22 females) emerged with an overall mean (±SE) of 7.2 ± 3.4 adults per bolt. Adults emerged from all 4 bolts in the largest diameter class (12.0 ± 5.9 adults per bolt) but from only half of the bolts in the 2 smaller size classes (2.4 ± 1.6 adults per bolt).

Development bioassays were initiated in May 2023 and continued until February 2026. Logs of each tree species were individually placed in pop-up cages made of fine mesh (≈0.2 mm) with 1 clear vinyl side (https://ecologysupplies.com, New Jersey, United States) together with fresh foliage of *U. americana* to provide food required for adult sexual maturation (Fig. 1D). *U. americana* logs collected from healthy trees with no evidence of *S. tridentata* infestation were used as controls. As for the feeding bioassays, American elm foliage was sampled from a tree growing near the AFC during summer or obtained from greenhouse-grown saplings during winter and placed in small water-filled containers to maintain leaf turgor. Foliage was replaced every 48 hours. One pair of adult *S. tridentata* (1 male and 1 female) was introduced into each cage. Females were allowed to oviposit until death. Females that died during the experimental period were dissected to verify the presence of fertilized eggs. If no fertilized eggs were detected, a new female (or a new pair, if both adults had died) was introduced. Only experimental units in which females survived for at least 10 days were considered valid and subjected to the experimental protocol. Once the female had died and the presence of mature eggs in the female abdomen had been confirmed, each log was maintained at room temperature for 3 months to allow larval establishment and development. Subsequently, the logs were kept at 5 °C for 2 weeks and then at −12 °C (or −20 °C, explained below) for 3 months to simulate overwintering. Finally, each log was returned to room temperature in the containment tulle cages for an additional 3 months to allow the possible emergence of adult insects (Flaherty et al. 2011). During the periods in which the logs were maintained at room temperature, they were sprayed 5 to 7 days per week with water to reduce the risk of desiccation. At the end of the exposure period, logs were debarked (Fig. 1E) and inspected for the presence of larval galleries and the number of dead larvae (Fig. 1F).

Experimental trials were conducted from June 2023 to May 2026. Due to the limited availability of adult insects, logs were exposed in 3 separate batches. The first batch included 23 logs (May to July 2023), the second included 25 logs (February 2024), and the third included 21 logs (March to June 2025). Due to an oversight the overwintering temperature for the third batch of logs was −20 °C instead of −12 °C. The total number of replicates per tree species is reported in Table 1. Elm species were generally prioritized based on information available in the literature; consequently, the number of logs varied among species. Nevertheless, each tree species was represented by at least 3 valid replicate logs that completed the entire experimental protocol. Log inspections began in February 2024 and concluded in May 2026.

**Table 1.** Number of log replicates, number of valid log replicates (ie experimental units in which a female survived for ≥10 days and eggs were confirmed after female dissection), total number of emerged adults and dead larvae, and mean ± standard error (SE) number of adults and dead larvae for each tested tree species (2023 to 2026)

Species	Replicates	Valid replicates	Adults (total)	Adults (mean ± SE)	Larvae (total)	Larvae (mean ± SE)
*U.*	10	5	0	–	48	9.60 ± 5.89
*U. glabra*	6	5	0	–	3	0.60 ± 0.40
*U. laevis*	9	3	0	–	14	4.67 ± 4.67
*U. minor*	7	7	3	0.43 ± 0.30	56	8.00 ± 1.38
*U. pumila*	10	5	0	–	6	1.20 ± 0.73
*A. campestre*	5	5	0	–	0	–
*A. platanoides*	5	3	0	–	0	–
*A. pseudoplatanus*	4	4	0	–	0	–
*P. alba*	4	3	0	–	0	–
*P. nigra*	4	3	0	–	0	–
*P. tremula*	5	3	0	–	0	–

### Statistical Analyses

To assess the feeding activity of *S. tridentata* adults after 24 and 48 hours, 4 models were fitted: 2 for males (1 for no-choice and 1 for choice trials) and 2 for females (1 for no-choice and 1 for choice trials). No-choice trials were analyzed using linear models, while choice trials (*U. americana* vs. European species within each cup) were analyzed using linear mixed models, with cup identity included as a random factor to account for the paired design. In all models, the response variable was the leaf surface area consumed (mm^2^), ln-transformed to improve residual linearity, and the categorical explanatory variable was the tree species. *U. americana* was set as the reference level for comparisons with European tree species. Because feeding assessments were not always recorded exactly at 24 and 48 hours, the exact number of hours elapsed from the start of the experiment to each measurement was incorporated in all models as a ln-transformed offset. All the analyses were run in R (R Core Team 2025). No statistical analyses were performed on development bioassays due to the low number of replicates (Table 1).

## Results

### Feeding Bioassays

*S. tridentata* fed on all European elm species tested, whereas no feeding activity was observed on the other tree species examined (ie *A. campestre*, *A. platanoides*, *A. pseudoplatanus*, *P. alba*, *P. nigra*, and *P. tremula*). Some replicates were lost at 48 hours due to leaf desiccation, which prevented accurate measurement of leaf area. Therefore, all desiccated replicates (including some *Ulmus* replicates) were excluded from the statistical analyses. In addition, all *Acer* and *Populus* species were omitted from the analyses because no feeding activity was observed.

**Fig. 2.**
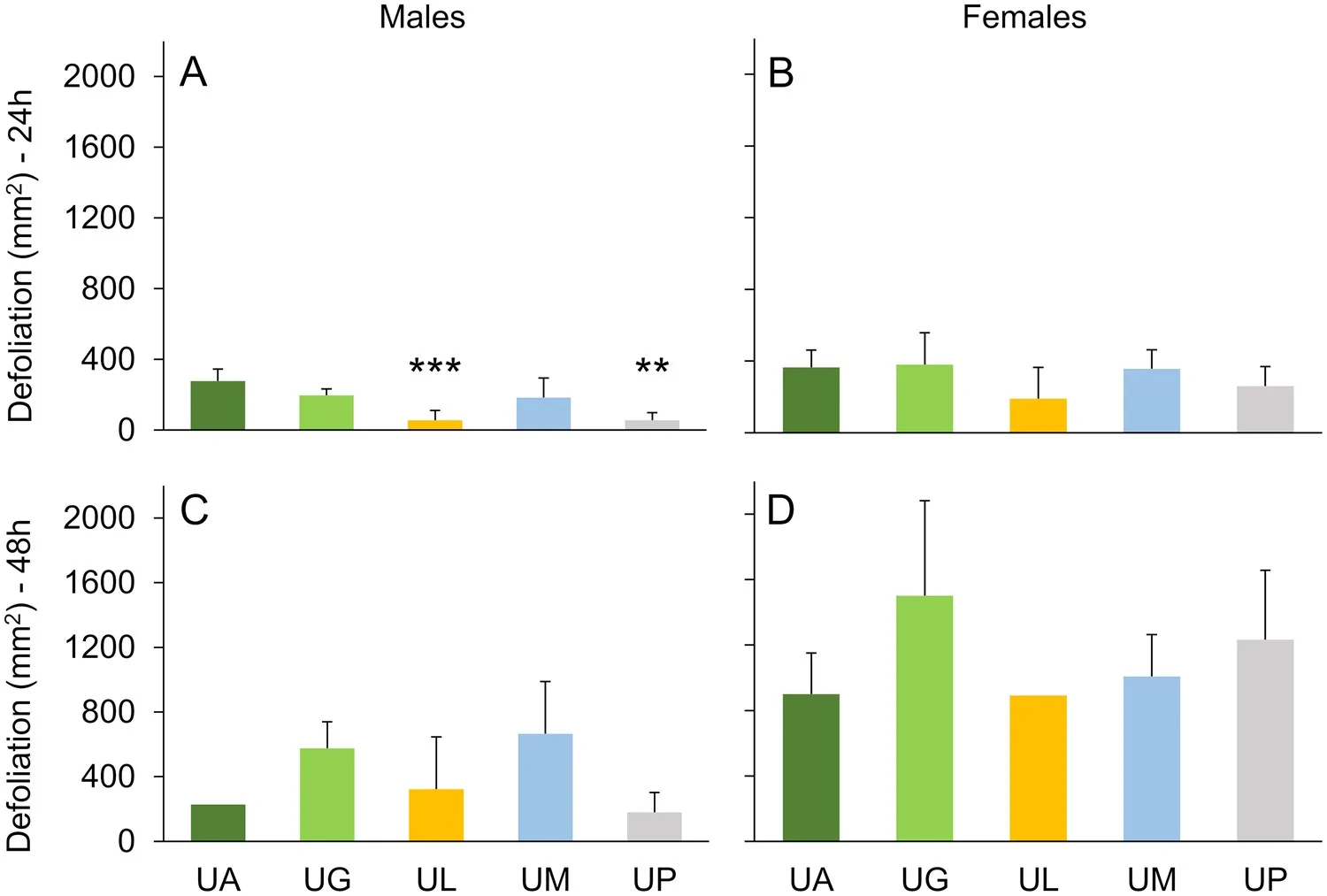
Feeding activity by *S. tridentata* males (A, C) and females (B, D) observed in no-choice bioassays at 24 hours (A, B) and 48 hours (C, D) after adult release in plastic cups containing leaf branches of *U. americana* and European elm species. Error bars represent positive standard errors. Asterisks indicate significant differences compared with *U. americana* from the linear models (*P*-value: * = 0.05–0.01; ** = 0.01–0.001; *** = < 0.001). Other tested tree species (ie *Acer* spp. and *Populus* spp.) are not shown because no feeding activity was recorded. UA, *U. americana*; UG, *U. glabra*; UL, *U. laevis*; UM, *U. minor*; UP, *U. pumila*.

In no-choice trials, feeding activity of *S. tridentata* males after 24 hours was significantly lower on *U. laevis* and *U. pumila* than on *U. americana* (control), while no significant differences were detected between *U. americana* and *U. glabra* and *U. minor* (Table 2; Fig. 2A). For females after 24 hours (Fig. 2B), and for both males and females after 48 hours (Fig. 2C and D), no significant differences in feeding activity were detected between *U. americana* and the other elm species (Table 2).

**Table 2.** Output of the linear models testing feeding activity (ln-transformed leaf surface area consumed, mm^2^/hour) of *S. tridentata* males and females after 24 and 48 hours, in no-choice trials

	Estimate	SE	*t*-value	*P*-value
**Males**				
***24 hours***				
	2.241	0.738	3.038	**0.006**
*U. glabra*	−0.216	1.143	−0.189	0.852
*U. laevis*	−4.332	1.143	−3.791	**<0.001**
*U. minor*	−1.344	1.143	−1.176	0.251
*U. pumila*	−3.690	1.086	−3.398	**0.002**
*F*_4,23_ = 5.887, *P*-value = **0.002**, R^2^ = 0.506				
***48 hours***				
Intercept	1.581	2.708	0.584	0.568
*U. glabra*	0.726	2.966	0.245	0.810
*U. laevis*	−4.008	2.966	−1.351	0.195
*U. minor*	−0.469	3.028	−0.155	0.879
*U. pumila*	−2.156	2.925	−0.737	0.472
*F*_4,16_ = 2.227, *P*-value = 0.112, R^2^ = 0.358				
**Females**				
***24 hours***				
Intercept	2.379	1.252	1.900	0.073
*U. glabra*	−1.708	1.770	−0.965	0.347
*U. laevis*	−3.316	1.770	−1.873	0.077
*U. minor*	−0.742	1.770	−0.419	0.680
*U. pumila*	−1.040	1.878	−0.554	0.586
*F*_4,19_ = 0.998, *P*-value = 0.433, R^2^ = 0.174				
***48 hours***				
Intercept	2.724	0.458	5.950	**<0.001**
*U. glabra*	0.308	0.647	0.475	0.644
*U. laevis*	0.199	1.024	0.195	0.849
*U. minor*	0.145	0.647	0.224	0.827
*U. pumila*	0.428	0.699	0.612	0.553
*F*_4,11_ = 0.112, *P*-value = 0.976, R^2^ = 0.039				

*U. americana* was used as the reference category (model intercept). Significant *P*-values (*P *< 0.05) are in bold. *F* statistics (with numerator and denominator degrees of freedom), its overall *P*-value, and R^2^ are reported for each model. SE, standard error.

**Fig. 3.**
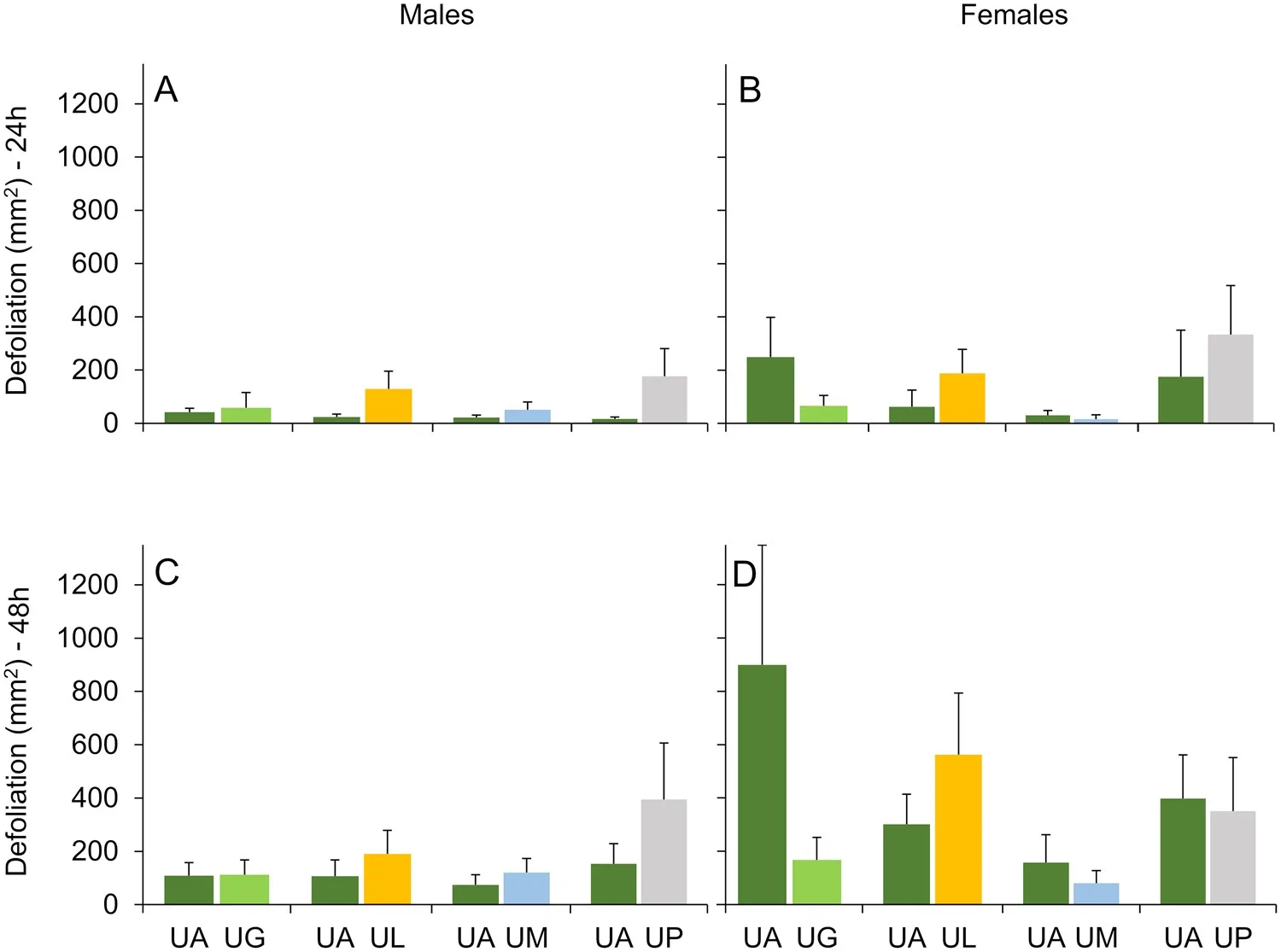
Feeding activity by *S. tridentata* males (A, C) and females (B, D) observed in choice bioassays at 24 hours (A, B) and 48 hours (C, D) after adult release in plastic cups containing leaf branches of both *U. americana* and one of the European elm species. Error bars represent positive standard errors. Other tested tree species (ie *Acer* spp. and *Populus* spp.) are not shown because no feeding activity was recorded. UA, *U. americana*; UG, *U. glabra*; UL, *U. laevis*; UM, *U. minor*; UP, *U. pumila*.

In choice trials, *S. tridentata* adults showed no feeding preference when given a choice between *U. americana* and the other elm species tested. No significant differences in consumed leaf area were detected among tree species at either 24 or 48 hours for either males or females (Table 3; Fig. 3). When *U. americana* was paired with *Acer* or *Populus* species, feeding occurred exclusively on *U. americana*, consistent with the results of no-choice trials.

**Table 3.** Output of the linear mixed models testing feeding activity (ln-transformed leaf surface area consumed, mm^2^/hour) of *S. tridentata* males and females after 24 and 48 hours, in choice trials

	Estimate	SE	df	*t*-value	*P*-value
**Males**					
***24 ***					
Intercept	−1.053	0.434	27	−2.424	**0.022**
*U. glabra*	1.200	1.034	24	−1.161	0.257
*U. laevis*	0.714	0.971	24	0.735	0.469
*U. minor*	−0.273	0.971	24	−0.281	0.781
*U. pumila*	0.543	0.921	24	0.589	0.561
LRT = 3.053, *P*-value = 0.549, mR^2^ = 0.049, cR^2^ = 0.049					
***48 hours***					
**Intercept**	−0.508	0.584	19	−0.871	0.395
*U. glabra*	−1.173	1.430	15	−0.820	0.425
*U. laevis*	1.660	1.617	15	1.027	0.321
*U. minor*	−0.284	1.147	15	−0.247	0.808
*U. pumila*	1.254	1.305	15	0.961	0.352
LRT = 3.484, *P*-value = 0.480, mR^2^ = 0.077, cR^2^ = 0.077					
**Females**					
***24 hours***					
Intercept	−0.953	0.590	19	−1.613	0.123
*U. glabra*	0.064	1.229	16	0.052	0.959
*U. laevis*	2.018	1.320	16	1.529	0.146
*U. minor*	−1.112	1.320	16	−0.842	0.412
*U. pumila*	2.271	1.446	16	1.570	0.136
LRT = 6.393, *P*-value = 0.172, mR^2^ = 0.135, cR^2^ = 0.135					
***48 hours***					
Intercept	0.932	0.602	17	1.546	0.140
*U. glabra*	−2.042	1.363	14	−1.450	0.156
*U. laevis*	0.227	1.240	14	0.183	0.857
*U. minor*	−1.751	1.240	14	−1.412	0.180
*U. pumila*	−0.396	1.363	14	−0.291	0.778
LRT = 4.380, *P*-value = 0.357, mR^2^ = 0.097, cR^2^ = 0.234					

*U. americana* was used as the reference category (model intercept). Significant *P*-values (*P *< 0.05) are in bold. Likelihood-ratio test (LRT; full vs. null model with same random structure), its overall *P*-value, and marginal (m) and conditional (c) pseudo-R^2^ are reported for each model. SE, standard error; df, degrees of freedom.

The mean (±standard error) elytra length and maximum thoracic width of retained specimens were 7.56 (±0.09) mm and 2.86 (±0.04) mm in males, and 8.71 (±0.17) mm and 3.57 (±0.17) mm in females, respectively.

### Development Bioassays

In the trials conducted from 2023 to 2026, 22 of the 23 logs in the first batch, 3 of the 25 logs in the second batch, and all 21 logs in the third batch were considered valid experimental units. All European elm species proved suitable for the larval development of *S. tridentata*, with a total of 127 larvae recorded from 46 valid log replicates (Table 1). The highest number of larvae (56) was recorded in *U. minor*. Of the 127 larvae recorded, 84 were obtained from the first batch of logs, none from the second batch, and 43 from the third batch. However, no adult insects emerged from any elm species, including *U. americana*, except for *U. minor*, from which 3 male individuals emerged from 2 logs in the first batch. None of the *Acer* or *Populus* species proved suitable for insect development.

## Discussion

The importation of elm wood from North America into the European Union inevitably entails a risk of introducing exotic wood-boring beetles, despite phytosanitary measures (Allen et al. 2017, Haack et al. 2022). In this context, the longhorn beetle *S. tridentata* has recently been identified as a potential phytosanitary threat to the European Union following repeated interceptions of larvae in logs with bark originating from several states in the eastern United States of America (Binazzi et al. 2019, EFSA et al. 2020). However, the limited information available on its host range, particularly with regard to tree species native to or widespread in Europe, has hindered a comprehensive assessment of its potential establishment and impact in the region. Our results indicate that *S. tridentata* can feed on leaves of all 4 tested European elm species but not on European maples and poplars. Similarly, larvae were found in all European elm species but not on tested maples and poplars. Together, these findings suggest that European elms can serve as suitable hosts for both adult feeding and larval development.

No-choice feeding bioassays showed that *S. tridentata* adults successfully fed on all tested elm species, whereas no feeding activity was observed on the tested poplar and maple species. Similarly, choice feeding bioassays showed that *S. tridentata* adults did not show a specific preference when forced to choose between *U. americana* and European elms but again avoided maples and poplars. These results may reflect different ecological mechanisms. First, the tendency of *S. tridentata* adults emerging from field-collected *U. americana* logs to feed on elm leaves is consistent with patterns reported for several cerambycid species, in which adults preferentially feed on the same host species or genus that supported their larval development (Hanks 1999, Hanks and Wang 2017). Second, differences in feeding activity among tree species are commonly reported in longhorn beetles, reflecting varying levels of host acceptance and suitability (Broberg and Borden 2005, Naves et al. 2006, Faccoli and Favaro 2016, Yasui et al. 2024). These differences are often associated with volatile compounds emitted by the host plants or with secondary metabolites and other chemical compounds contained in the leaves, which can either stimulate or deter feeding behavior (Morewood et al. 2004, Miller and Ware 2022). For poplars, this avoidance may be linked to the presence of salicinoids in the leaves, a group of specialized phenolic compounds produced by plants in the Salicaceae family (Boeckler et al. 2011). These compounds are known to affect insect herbivores by reducing food consumption and impairing digestion and have been shown to exert a deterrent effect in other longhorn beetle species (Mason et al. 2021). In maples, a similar effect may be mediated by terpene hydrocarbons, which are abundant in certain *Acer* species and can also deter insect feeding behavior (Loughrin et al. 1997). Although these patterns suggest a role of host-related chemical cues in shaping feeding behavior, the limited space available in the choice experiments may have influenced insect responses, as beetles were exposed to cues from 2 different host species within the same cup.

Development bioassays showed that larvae were present in logs of all tested European elm species, whereas no larvae were recorded in the tested poplar and maple species. Host-dependent variation in larval survival and development has been reported for several cerambycid species (Hanks et al. 1999, Morewood et al. 2003, Yan et al. 2008, Mazaheri et al. 2011, Urano et al. 2022, Yan et al. 2022), suggesting that host suitability plays a key role in determining successful larval establishment. Such differences are likely associated with variation in the physical and chemical properties of host tissues, including wood density, moisture content, bark thickness, and the concentration of defensive compounds such as phenolics and terpenoids (Paine et al. 2000, Hayes et al. 2014, Nahrung et al. 2014, Torres et al. 2024, Osowiecki and Keena 2025). Although the mechanisms underlying the observed differences among the tested species remain unknown for *S. tridentata*, the exclusive occurrence of larvae in elm logs suggests that elm species provide more suitable conditions for larval establishment and development than poplar and maple. This pattern may reflect the closer taxonomic and physiological similarity of European elm species to the beetle’s primary hosts in its native range (eg *U. americana*). However, our experimental design did not allow us to assess oviposition rates among the tested host species, nor to quantify larval mortality during development. Therefore, the observed absence of larvae in maple and poplar logs may result from differences in host acceptance by females, larval establishment and survival, or a combination of both. Additional studies are needed to disentangle these mechanisms and to more accurately determine the relative suitability of the tested host species.

We also found that only 3 adult beetles emerged from the logs despite the high number of larvae recorded within the wood. Notably, all 3 adults emerged from *U. minor*. Previous studies on longhorn beetles have shown that host suitability may influence not only larval establishment but also the successful completion of development (Paine et al. 2000, Bancroft et al. 2002, Faccoli and Favaro 2016). However, the very low adult emergence observed in this study should not be interpreted as evidence of poor host suitability. In fact, no adults emerged from *U. americana* under the experimental conditions we adopted, despite this species being considered a primary host of *S. tridentata*. This finding contrasts with the high emergence rates observed in the preliminary trial conducted in 2022 and suggests that factors other than host suitability may have limited adult emergence. One possible explanation is the progressive desiccation of the logs. Despite efforts to reduce water loss, including sealing the cut ends with paraffin and spraying with water, many logs showed signs of substantial desiccation. Reduced moisture content may have negatively affected the survival of larvae developing beneath the bark, where the youngest instars feed before penetrating deeper into the wood. It is also possible that the transition in temperatures when we overwintered the logs were not ideal for larval survival. Although *S. tridentata* larvae are adapted to survive freezing temperatures in North American winters (eg the mean minimum monthly temperature measured at the Fredericton International Airport, New Brunswick, during December to February, from 2023 to 2026, averaged −11.7 °C [Environment Canada]), the drop in temperature from 5 °C to −12 °C or −20 °C, when logs were placed in the freezer for overwintering, may have been too sudden to allow larvae to fully acclimate.

## Conclusions

Our findings are important from both a biological and an invasion ecology perspective. From a biological standpoint, our results further support the close association of *S. tridentata* with elm species. In contrast, despite historical records reporting *Acer* and *Populus* as hosts, we found no evidence of feeding or larval development on the tested maple and poplar species. These tree genera should therefore not be considered potential hosts in pest risk analyses. From an invasion ecology perspective, the widespread distribution of elm trees across Europe may provide suitable conditions for the establishment of *S. tridentata* should the species be introduced. This possibility is further supported by the ongoing decline of many European elm populations due to Dutch elm disease. Because *S. tridentata* preferentially colonizes stressed, declining, or recently weakened trees (Campbell et al. 1989), the abundance of disease-affected elms may increase the availability of suitable breeding material and thereby facilitate the establishment and spread of the beetle in invaded areas. Future studies should further investigate the developmental success of *S. tridentata* on European host species and include individuals originating from different parts of the species’ native range. Indeed, host-use patterns may vary among geographically distinct populations, and therefore the possibility that some populations are capable of feeding and developing on maple or poplar species cannot be excluded, although it appears unlikely.

## Data Availability

The datasets generated and analyzed during the current study are available from the corresponding author on reasonable request.
